# Interplay between Gut Lymphatic Vessels and Microbiota

**DOI:** 10.3390/cells10102584

**Published:** 2021-09-28

**Authors:** Eleonora Solari, Cristiana Marcozzi, Daniela Negrini, Andrea Moriondo

**Affiliations:** Department of Medicine and Surgery, School of Medicine, University of Insubria, 21100 Varese, Italy; eleonora.solari@uninsubria.it (E.S.); cristiana.marcozzi@uninsubria.it (C.M.); daniela.negrini@uninsubria.it (D.N.)

**Keywords:** lymphatic vessel, lacteal, microbiota

## Abstract

Lymphatic vessels play a distinctive role in draining fluid, molecules and even cells from interstitial and serosal spaces back to the blood circulation. Lymph vessels of the gut, and especially those located in the villi (called lacteals), not only serve this primary function, but are also responsible for the transport of lipid moieties absorbed by the intestinal mucosa and serve as a second line of defence against possible bacterial infections. Here, we briefly review the current knowledge of the general mechanisms allowing lymph drainage and propulsion and will focus on the most recent findings on the mutual relationship between lacteals and intestinal microbiota.

## 1. Foreword

The lymphatic system is a fascinating and still partially undiscovered fluid transport system that lies in parallel with the blood circulation and complements it by returning the liquid filtered from the blood capillaries towards the interstitial spaces back to the blood stream. Its role is fundamental in maintaining a functional fluid volume and composition in various areas of the body, preventing organ failure. In this review, we will briefly discuss the general mechanisms of lymph drainage and propulsion, and then focus on the most recent findings that pertain to the exquisite, peculiar environment of the initial lymphatic vessels of the gut, the lacteals. They have recently been the site of extensive research because of the pivotal role that the close association between lacteals and microbiota exerts on the whole-body homeostasis.

## 2. General Overview of the Lymphatic System

**Figure 1:**
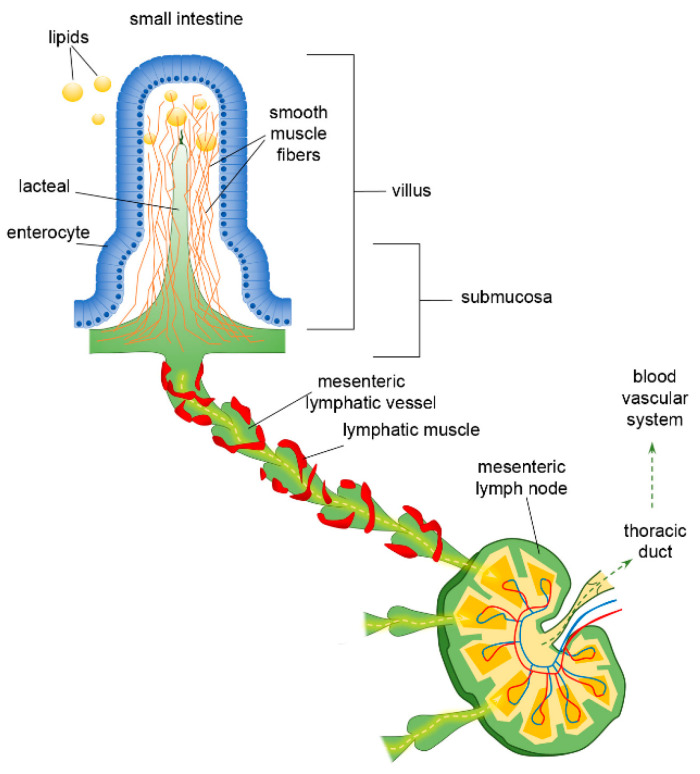
Functional organisation of lymphatic capillaries (*lacteals*), submucosal and mesenteric collecting vessels in the intestine. Dietary lipids are absorbed at the epithelial surface of the intestine, entering lacteals by paracellular and/or transcellular mechanisms. Lacteals are located at the centre of the intestinal villus, surrounded by villus smooth muscle fibres. They merge at the villus basis forming the submucosal network and then lymph is propelled along mesenteric collecting vessels endowed with a lymphatic muscle mesh. Lymph passes through mesenteric lymph nodes, ultimately reaching the venous circulatory system via the thoracic duct.

The lymphatic vasculature plays a crucial role in maintaining tissue fluid balance, draining fluid, plasma macromolecules and cells (the so called “*lymph”*) from the interstitial tissues and serosal cavities. Moreover, it is critically involved in immune surveillance by antigens and immune cells trafficking and in conveying dietary lipids from the intestine, returning them back to the circulatory system [1,2,3]. The lymphatic network is widely distributed throughout the body, arising as lymphatic *capillaries*, thin-walled vessels devoid of lymphatic muscle (LM). They display a discontinuous basement membrane and a single layer of overlapping endothelial cells (LECs), which form flap-like openings defined as the primary valve system [4,5]. Anchoring filaments connect LECs to the extracellular matrix, allowing the valve opening to favour the one-way entrance of interstitial fluid, cells, proteins, chylomicrons as well as pathogens, also avoiding vessel collapse at higher interstitial pressure [6]. Furthermore, primary valves closure prevents reverse lymph leakage into the surrounding interstitium [4]. Lymphatic capillaries then drain into progressively larger and converging *collecting* lymphatics (Figure 1). Their vessel’s wall is equipped with an LM layer owning unique features (as it displays skeletal, cardiac, and smooth muscle contractile elements) [7] and possesses a continuous endothelial layer, thus avoiding lymph outflow. Collecting lymphatics typically exhibit intraluminal valves [8], which allow the unidirectional lymph progression along the network and separate adjacent vessel segments named “*lymphangions*”, the functional contracting pump units of the lymphatic system. By acting as a sequential chain of pumps, lymph fluid is propelled towards lymph nodes and, eventually, in the thoracic duct or in the right lymphatic duct, emptying into the blood venous circulatory system [9]. Although in many body areas, lymphatic vessels mainly provide a route for water and molecules filtered from blood vessels, mesenteric lymphatics mostly collect fluid and lipids coming from the intestinal lumen. Dysfunctional lymphatics and/or inadequate valve development result in tissue fluid accumulation (named “*lymphedema*”), chylous ascites, chylothorax, inflammation and metabolic disorders [10,11,12].

The proper function of the lymphatic system is critically related to the development of pressure gradients between the vessel’s segments and/or surrounding tissue. According to Starling’s Law [1], lymph formation depends upon the transmural pressure gradient (Δ*P_TM_*) between intraluminal (*P_Lymph_*) and interstitial (*P_int_*) hydraulic pressures (Δ*P_TM_ = P_Lymph_* − *P_int_*). Lymph propulsion is due to the intraluminal hydraulic pressure gradient (Δ*P_Lymph_*) across adjacent lymphangions (Δ*P_Lymph_ = P_L,_*_1_ − *P_L,_*_2_), acting against an overall opposite pressure gradient [13]; in most tissues’ lymphatic capillaries, *P_Lymph_* is almost slightly subatmospheric [1], whereas in the venous system, the intraluminal pressure is ~10 cmH_2_O. However, exceeding the transvalve Δ*P_Lymph_* (1–1.5 cm H_2_O) is enough to guarantee the proper lymph propulsion to the downstream lymphangion, against an adverse hydraulic pressure gradient and the force of gravity [13].

Δ*P_TM_* and Δ*P_Lymph_* are deeply affected by different mechanisms, either involving the spontaneous contraction of the vessel itself (“intrinsic” mechanism) or originating in the surrounding tissues (“extrinsic” mechanisms). The intrinsic mechanism is predominant in vessels located in soft tissues and body areas experiencing no significant tissue displacement, such as mesenteric lymphatics. It relies on spontaneous contractions of the vessel triggered by pacemaker cells in the LM layer [14,15] and then transmitted to electrically coupled LM cells in the vessel’s wall [9]. Different pacemaking mechanisms have been proposed, such as Spontaneous Transient Depolarisations (STDs) [16,17] induced by calcium-dependent chloride currents or I_f_-like currents. The latter consist of an inward current that slowly activates on hyperpolarisation, due to hyperpolarisation-activated cyclic nucleotide (HCN) channels, similarly to what occurs in the heart sinoatrial node [18,19]. Hence, in analogy to the cardiac cycle, LM intrinsic activity generates phasic contractions, displaying an active systolic phase, which forces lymph propulsion to the adjacent vessel segment, and a passive diastolic phase, due to LM relaxation, which favours lymphangion fluid refilling. The whole mechanism can be described in terms of contraction frequency and ejection fraction or stroke volume [12,20]. Lymph flow dynamics and the surrounding microenvironment can deeply affect lymphatic spontaneous contractions. Changes in transmural and/or intraluminal pressures, lymph flow-induced wall shear stress, nitric oxide (NO), histamine, fluid osmolarity, local tissue temperature and neuronal modulation by the autonomous nervous system can significantly alter contraction frequency (i.e., chronotropic effect) and/or contraction amplitude (i.e., inotropic effect), continuously modulating and adapting lymph drainage and transport to current needs [21,22,23,24,25,26,27,28,29,30,31,32]. Impaired intrinsic contractility, as well as lymphatic vessels obstruction, may lead to oedema development as a result of tissue fluid imbalance [1].

The extrinsic mechanism, on the other hand, is related to mechanical stresses arising in surrounding tissues then transmitted to the lymphatic vessels by means of fibrous elements of the extracellular matrix [6]. It typically involves vessels located in areas of the body which experience cyclical movements such as the heart or skeletal muscle, lymphatics undergoing cardiogenic activity or respiratory movements, intestinal motility, external compression and arteriolar vasomotion [23,33,34,35,36,37,38]. These mechanisms rhythmically exert external forces compressing and expanding lymphatic vessels, thus dramatically affecting primary and intraluminal valves dynamics and both Δ*P_TM_* and Δ*P_Lymph_*.

Intrinsic and extrinsic mechanisms may coexist according to area on the body: their relevance depends upon the sources of extrinsic forces ranging from blood vessels’ vasomotion caused by the pulsatile blood flow, to skeletal muscle fibres’ contraction. Indeed, in the rat diaphragmatic lymphatic network, both intrinsic and extrinsic mechanisms cooperate as the contraction of the skeletal muscle fibres is adequate to sustain lymph flow in vessels of the tendinous and medial muscle regions, but it is not sufficient in the muscular periphery adjacent to the chest wall, where intrinsic contractions are required to prevent fluid accumulation [39,40,41]. However, if extrinsically related mechanisms are sufficient to generate lymph flow-supportive pressure gradients for proper lymph propulsion, when flow rates are elevated, lymphatic vessels generally display their own flow-induced inhibition of the spontaneous contractions, and lymphatic vessels behave like conduits [42].

## 3. The Lymphatic System of the Intestine and Mesentery

The organisation of the lymphatic network greatly varies among different body areas. In the intestine, a three-level distribution of lymphatic vessels can be identified: (a) in the small intestinal villi, (b) in the submucosa and (c) in the smooth muscle layer surrounding the mucosa [43]. The blind-ended lymphatic capillaries, also known as intestinal *lacteals* (Figure 1), are exclusively located in the centre of villi, normally reaching 60–70% of the villus length [44], which is, however, variable among different intestine tracts. Indeed, villi length decreases from the duodenum to the jejunum and distal ileum As a result, according to the absorptive properties of the intestinal epithelium, lacteals are longest in the duodenum, where most nutrient uptake occurs. Lacteals merge at the villi basis, forming the submucosal network. Intestinal villi contain a blood vascular capillary network and 1–10 central lacteals, providing a route for absorbed nutrient distribution [45,46]. Water-soluble molecules enter blood vessels and are transported to the portal vein; conversely, lipids and other lipophilic molecules of large size such as chylomicrons enter lymphatic vessels, which then reach the blood circulatory system without passing through the liver. Such a privileged delivery route can also be used to enhance the bioavailability of oral lipophilic drugs, thus improving the efficacy of therapeutical strategies [47,48]. As with other lymphatic capillaries, lacteals are non-contracting vessels, having no LM elements in their vessels’ walls nor intraluminal valves. Therefore, lymph drainage by intestinal lymphatics is deeply affected by extrinsic forces related to vasomotion and intestinal motility [49,50]. Indeed, the pulsatile activity of neighbouring arteries as well as villous motility may easily mechanically deform lymphatics. Lacteals are surrounded by villus smooth muscle fibres, organised in a tree-like structure (Figure 1), whose contractile activity exerts extrinsic forces contributing to enhance intestinal lymph and blood flow, propelling lymph at velocities up to 150 µm/s [51], with a positive effect on lipid absorption [50]. Lacteals’ periodic squeezing due to the contraction of those longitudinally oriented smooth muscle fibres is critically modulated by neurohormonal factors released by the autonomic nervous system. Thus, in the intestinal lymphatic network, neuromodulation may exert a mixed modulatory role by acting on both intrinsic and extrinsic mechanisms of drainage and propulsion [51]. Moreover, the contraction of smooth muscle layers in the intestinal wall gives rise to a compressive stress on lacteals and gut lymphatics, favouring vessel squeezing and lymph propulsion. On the contrary, when smooth muscle relaxes, lymphatics are stretched and a net Δ*P_TM_* and/or Δ*P_Lymph_* favouring fluid entry is provided. Thus, intestinal lymph drainage and propulsion are pulsatile. Lymphatic vessels in the smooth muscle layers are anatomically segregated from submucosal ones; however, both networks merge into larger collectors next to the mesentery, where almost all the lymph is of intestinal origin [52]. Here, the collecting vessels are equipped with intraluminal valves and a proper LM mesh (Figure 1) so that intrinsic spontaneous contractions can be identified along the lymphangion chain, allowing lymph propulsion. In rat mesenteric lymphatics, spontaneous contractions arise in the smaller vessels and then propagate to the larger collecting lymphatics, generating progressively higher pressure oscillations from distal (2–4 cmH_2_O) to proximal vessels (up to 10–20 cmH_2_O) [13]. Those lymphatics display an intrinsic contraction frequency of about of 6.4 ± 0.6 cycles/min and an ejection fraction of about 67% of their resting diastolic volume [53]. Lymph propelled along the mesenteric lymphangions chain passes through mesenteric lymph nodes, then drains into the thoracic duct and, eventually, empties into the blood circulatory system at the level of the subclavian vein (Figure 1).

The proper development of a fully functional lymphatic system, essential to guarantee fluid homeostasis, is critically related to the master regulatory gene Prox1 (Prospero homeobox protein 1), as Prox1-null mice are devoid of lymphatic vessels, whose deficiency results in severe oedema and prenatal death at embryonic day E14.5 [54]. Heterozygous Prox1 mice often die at birth or soon after birth, mainly due to lymphatics’ defective growth, particularly displaying dilated and dysfunctional submucosal and mesenteric vessels, and impaired lymph drainage, also resulting in chylous ascites and/or chylothorax [55]. However, in surviving haplo-insufficient mice lymph, abnormal leaking from gut lymphatics in the visceral area accumulates in the surrounding tissues and causes an increase in adipose tissue. This results in adulthood late-onset obesity due to subcutaneous and intra-abdominal dysfunctional lymphatic-related fat accumulation and, eventually, adipocyte proliferation [55,56]. Moreover, VEGFC (Vascular endothelial growth factor C) growth factor, which is implicated in prenatal lymphatic system development, is also required for intestinal lymphatics’ maintenance during adulthood [57]. As VEGFC-null mice die around embryonic day E15.5–17.5 lacking lymphatic vessels differentiation, postnatal deletion of VEGFC results in the regression of lacteals. Since, in adulthood, lacteals continue to grow and expand to guarantee proper lipid absorption, smooth muscle fibres located in the villi and within the intestinal inner circular muscle layer may be the prominent VEGFC source to maintain proper organisation of intestinal lymphatics [57,58].

## 4. The Peculiar Environment of the Lacteal–Epithelial–Mucus Complex

In the 1990s, the gut lymphatic system gained attention as a primary route for the dissemination of pathogens, pathogen-derived toxins and, later, also tissue-derived proinflammatory mediators, which gave rise to multi-organ dysfunction syndrome (MODS). Even before that time, MODS was known to be caused by alterations in the gut mucosa integrity and consequent dissemination of inflammatory molecules, pathogenic bacteria, antigens and pancreatic-derived digestive enzymes, collectively referred to as “toxic” agents, to the rest of the body, usually starting from the lungs [59,60].

From a purely phenomenological view, the lacteals wall is not able to selectively avoid the drainage of pathogens, endotoxins and/or pro-inflammatory molecules present in the villi interstitial space. This is due to button-like junctions between adjacent LECs, which allow their free, overlapping ends to open and behave similar to unidirectional (primary) valves, favouring interstitial liquid (and all the dissolved and suspended particles) progression into the vessel lumen. Therefore, the prevention of lacteal-draining toxic gut-derived lymph to the rest of the body depends on the maintenance of mucus and epithelial cells’ integrity. The mucus layer protects the epithelium from digestive enzymes, which can damage this barrier by degradation of E-cadherin and TLR4 (Toll-Like Receptor 4) [61]. In healthy individuals, the gut microbiota produces short-chain fatty acids, which stimulate the epithelial cells to produce mucus and antimicrobial peptides, thus increasing the mucosal immune response. Mucus creates a favourable environment, which harbours commensal microbiota, protecting the intestine against colonisation by pathogenic agents [62], and a very hostile environment for pathogens, which are mostly excluded from reaching the epithelial layer [60,63]. In this situation, only commensal microbes could eventually be drained in the event of an increased epithelial permeability. Nevertheless, most evidence suggest that lymph drained from the gut in normal and in mice subjected to ischemia-reperfusion injury is sterile [59,60]. Despite the healthy intestine being lined by a monolayer of epithelial cells, it represents a proper selective barrier, thus controlling the movement of different substances and macromolecules. This is due to tight junctions (TJs) and junctional adherens molecules (JAMs) on the apical surface of the epithelium between neighbouring cells, forming a strong seal which regulates the paracellular pathway and prevents the uncontrolled systemic spread of potentially toxic agents [64,65]. In critical illness, TJs homeostasis can be impaired by proinflammatory cytokines, pathogens and lipopolysaccharides, damaging the integrity of the intestinal epithelium. The increase in barrier permeability with the loss of functionality affects not only fat absorption, but also leads to dysbiosis and to an inflammatory-related alteration of immunosurveillance. The regulated apoptosis of intestinal senescent epithelial cells, which are constantly replaced by new ones, represents an additional critical process to further maintain barrier integrity. Dendritic cells (DCs) are involved in the crosstalk signalling between the commensal microbiota and the epithelial host cells, regulating the balance between tolerance and active immunity to commensal microorganisms, leading to the appropriate physiologic inflammation and controlled apoptosis. DCs constantly sample intestine lumen, by monitoring dietary antigens and pathogens, and present antigens to lymphoid tissue where they stimulate the host immune response [66,67]. Epithelial cell loss due to bacterial- and/or inflammatory-enhanced apoptosis is therefore a key passage that leads to bacteria drainage and dissemination through the lymphatic system to the rest of the body. This situation could be detrimental even for non-harmful bacteria of the mucous layer.

## 5. Maturation and Stability of Lacteals

Lymphatic capillaries drain liquids and solutes from the surrounding interstitium thanks to the vessels’ thin wall made by a single LECs layer. Although it still remains controversial whether smooth muscle fibres found by many groups in the inner portions of the villi belong to the lacteal wall or not [44,68,69,70,71], the length and diameter of the lacteal provide an estimate of its potential drainage capability. In fact, the extent of the surface area exposed to the interstitial space determines the lymph flow attainable inside the vessel itself. Thus, during development, lacteals must extend the draining surface exposed to the villus interstitial space to allow proper liquid and dietary lipids entry. Simultaneously, lacteals also provide a second line of defence against pathogens entering the mucosal epithelium. They let submucosal DCs engulfed with captured bacterial cells to be transported to the first mesenteric lymph node before activating an immune response that, in normal subjects, remains compartmentalised to the prenodal district of the mesenteric lymphatic tree [72,73]. However, the so called “lymph-based bacterial translocation” theory suggests the possibility that free pathogens in the gut lymph may escape the lymph node control and spread to the rest of the body [74,75]. Therefore, lacteals’ maturation is subjected to two different, and paradoxically opposing, driving strategies. Considering their function as lipid-translocating vessels, the permeable surface must be maximised. Indeed, chylomicron entry is due to passive diffusion through lacteal primary valves (paracellular transport) and through a cell-mediated one (transcellular pathway). Both processes require a wider wall surface to be properly exerted. On the other hand, if we consider their role as a second line defence system against pathogens, passive drainage is less important. Indeed, DCs, as with other lymphoid cells, roll their way inside lacteals in an active, “intentional” process, not strictly dependent upon fluid flow. Considering the possible danger arising from an uncontrolled spread of free pathogens through mesenteric lymphatics, up to the thoracic duct and subclavian vein, lymph drainage could theoretically be avoided altogether. Therefore, the final shape and functionality of adult lacteals need to take into account these different driving principles.

To date, few mechanisms have been elucidated regarding the development and maintenance of a fully functional lacteal network in the adult subject, and, surprisingly, they all require the presence of a normal gut microbiota.

Lymph drainage from the interstitial space of the villi represents a balanced mechanism of different needs: while a lymph drainage increase can improve the immune surveillance keeping pathogens under control [76], on the other hand, a lymph drainage reduction can prevent damages caused by the spread of pathogens and/or pro-inflammatory factors coming from nutrients hydrolysis closely in contact with a deteriorated intestinal epithelium [59,77,78].

Lacteals sprout into the villi around postnatal day 7 and continue to develop and remodel after weaning at P21 (in mice), into adult life [79]. The first evidence of the need of gut microbiota for proper lacteals development came from the findings that germ-free (GF) mice, which entirely lack an endogenous microbiota, have decreased lacteal length and a significantly lower number of lymphatic endothelial cells (Prox1^+^) in their villi and reduced VEGFR3 (Vascular endothelial growth factor receptor 3) expression, when compared to same-age mice grown in a controlled, specific pathogen-free condition [80]. Disruption of intestinal lymphatics, in adult mice, leads to immune homeostasis failure and results in rapid lethality, due to the lack of immune surveillance that lacteals and mesenteric lymph nodes are expected to deploy [44,81]. Interestingly, lymphatic regression only affects lacteals, since this phenomenon was not observed in other organs and tissues where lymphatic networks are present, such as diaphragm, skin and trachea.

Gut microbiota is also continuously evolving in its composition from the early postnatal days until weaning and thereafter. It could then be plausible that not only its presence in the gut but also the evolution of its composition are necessary for lacteals’ normal development into their adult form. Indeed, mice of different postnatal ages between P7 (lactation) and P28 (after weaning) treated with an antibiotic cocktail showed a different response in terms of lacteals development and overall function depending on the pre- or post-weaning age. Until P14, well before weaning, no difference was found in lacteals length between wild type and antibiotic-treated mice, whereas only in mice older than P28 was there a significant lacteal shortening in antibiotic-treated mice with respect to wild type. This phenotype closely resembles that seen in GF mice. Moreover, the extent of lacteals development was different among distinct intestinal segments, jejunum and ileum being the most affected by the presence of microbiota, as opposed to duodenum [80]. As duodenum is the least densely populated tract of the small intestine [82], data confirm that both microbiota composition and density are responsible for proper lacteal development.

At the ultrastructural level, depletion of microbiota in GF or antibiotic-treated mice also affected the junctional structures between adjacent LECs. As stated above, normal functional junctions are button-like, but in treated mice, they decreased in favour of zipper-like junctions, which prevent adjacent cells from moving apart and opening the unidirectional flap-like valve characteristic of absorbing lymphatic capillaries [80]. Acting together, a more impermeable vessel wall and a less extended lacteal network cause a significant reduction in lipid drainage from the gut, as witnessed by experiments where fluorescence-labelled lipids were used as tracer molecules to follow their path in the mesenteric lymphatic network. However, lipid transport is not completely abolished in these conditions, since chylomicrons could always enter lacteals by means of the transcellular pathways involving caveolae. A more conclusive result came from the dosage of triglycerides and free fatty acids in the plasma of wild type and treated mice, where the latter exhibited a marked decrease in the plasma concentration of both lipids [80]. The tighter transformation of lymphatic junctions from button-like into zippers in intestinal lacteals, prevents fat uptake, thus preventing diet-induced obesity [65,83]. This evidence suggests that endothelial cell junctions are dynamic structures essential for chylomicron uptake by lacteals. However, several authors have discovered a strict link between defective lymphatics (mesenteric but also at the whole-body level) and fat accumulation and/or obesity [55,84]. Moreover, lipid-rich lymph stasis and fat accumulation in the intestinal wall might also be one of the major contributors to Crohn’s disease [85,86,87]. On the other hand, zippering junctions in lymphatics could also impair uptake of essential nutrients or compromise, in some way, fluid drainage and immune cell trafficking, leading to possible adverse effects.

Lymphangiogenesis is active during the early stages of development. However, the adult vasculature remains in a sort of quiescent, growth-factor-insensitive state when specific drainage needs are met. Adult lacteals’ endothelial cells continue to display filopodia and are positive for Ki67 proliferation markers even in adult subjects. Both pieces of evidence are signs of a proliferative and potentially remodelling-prone state in adult life also [5,88,89,90,91]. In fact, if GF animals or antibiotic-treated mice are converted to a normal microbiota phenotype in adulthood, lacteals length, Prox1 and VEGFR3 expression levels and lymphatic endothelial cells junctional types are reverted to typical pathogen-free mice counterparts [44]. This implies that lacteals have an intrinsically high level of plasticity to adapt and respond to the continuous variation of the gut microbiota.

Is the influence of the microbiota’s density and composition on lacteals development and stability a one-way relationship or is there a mutual exchange and effect by lacteals as well? While the DC-mediated transport of invading intestinal bacteria is well acknowledged, very few research studies are related to the possible alteration of the microbiota in response to a primitive impairment of lymphatic function. Among others, in chronic colitis mice, the supplementation of VEGFC causes an increase in lymph drainage from the small intestine, and this, in turn, alters the composition of the intestinal microbiota, causing a net reduction in its amount but not in its diversity. Overall, an increased Bacteroidetes/Firmicutes ratio caused by increased lymphatic drainage closed the gap towards a healthy microbiota profile, thus reducing colitis [92].

Despite the very small amount of data collected so far, it is envisaged that a more efficient lymphatic drainage might exert a positive effect on the composition of gut microbiota, potentially through a better immunological control on the phyla.

## 6. Molecular Players in the Microbiota–Lacteal Relationship

**Figure 2:**
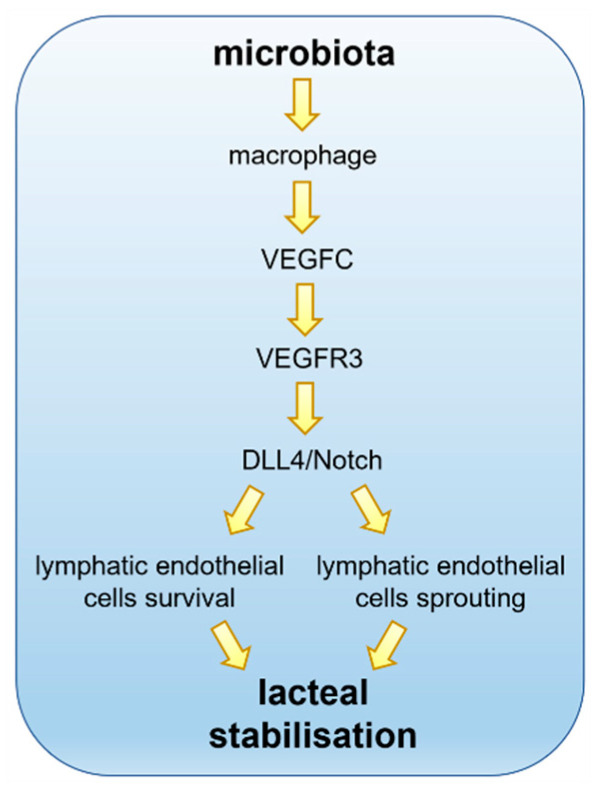
Lacteals in adult small intestinal villi are constantly remodelling. The gut microbiota interacts with intestinal villi and, despite the pivotal role played in absorption and transport of dietary lipids, the intestinal lymphatics are critically involved in the immune surveillance. The VEGFC pathways via VEGFR3 promote lacteals Notch signalling through DLL4 expression. Continuous Notch expression is required to promote lymphatic endothelial cells’ survival and sprouting, thus assuring lacteals stabilisation, optimal length maintenance and guaranteeing proper functionality.

As for other lymphatic networks in the body, the proper differentiation and proliferation of LECs is driven by Prox1 expression and by VEGFC–VEGFR3 signalling. Filopodia are one of the cytological markers of actively sprouting lymphatic vessels, maintained by lacteals also in adulthood. In this context, Notch signalling plays a controversial role, since Notch blockade has been seen to both promote and inhibit lymphatic sprouting. Overall, it might exert a site-specific effect since lymphatics in different areas of the body do not respond in the same manner to the signalling cascade mediated by Notch. In blood endothelial cells, a Notch ligand, DLL4 (Delta-Like Canonical Notch Ligand 4), is involved in vessel proliferation. In villi, high levels of DLL4 expression are present in the endothelial cells located at the sprouting tip of lacteals, and also, to a lesser extent, in the stem of the lacteal vessel (Figure 2). Additionally, Notch expression levels are high in lacteals. Thus, the steadily high level of Notch signalling could explain the continuous proliferative state of adult lacteals not common in the lymphatic networks localised elsewhere in the body. To strengthen this hypothesis, in transgenic mice where DLL4 expression was lowered, lacteals were significantly less developed than in wild type mice, but this difference was not present in the lymphatics of the skin [44].

In turn, DLL4 expression in lacteals endothelial cells is promoted by VEGFR3 signalling activated by VEGFD and VEGFC. Besides these phenotypical differences of the lacteals, blocking VEGFC/VEGFR3-DLL4 signalling also impairs lipid drainage and chylomicron transport along the mesenteric lymphatic network, resulting in a decreased concentration of fatty acids and triglycerides in the plasma of DLL4 knocked-out mice (Figure 2).

From this perspective, the source of VEGFC around lacteals is likely responsible for the overall homeostasis of lymphatic drainage and function. It is well known that smooth muscle cells of the villi, while performing a cyclic contraction–relaxation activity in order to induce the above mentioned “extrinsic pumping” mechanism for lymph formation and propulsion, also release VEGFC, maintaining lacteals’ integrity and thus proper lymph drainage function. However, given that the presence of intestinal microbiota has proven to be a key factor for the normal development and maintenance of adult lacteals, can it also be implied in VEGFR3/Notch/DLL4 signalling? Transgenic mice harbouring an inducible deletion of VEGFR3 show a lacteals phenotype closely resembling the one of GF mice, thus reinforcing this hypothesis. The same limited growth and maintenance of lacteal structure can be envisaged if VEGFC levels are low in the villus microenvironment. Such low levels of VEGFC can be detected in the intestinal tissue of both GF and antibiotic-treated mice, where gut microbiota content was found to be absent or low [80]. As expected, when GF mice were reverted by faecal transplant to a normal microbiota phenotype, VEGFC expression levels in the gut were also restored to the ones of pathogen-free mice. The expression level of none of the other major determinants of lymphangiogenesis seemed to be affected by microbial depletion [93,94,95,96,97]. The most obvious source of VEGFC in the villi is represented by smooth muscle cells, so the possible link between gut microbiota levels and their VEGFC production remains to be elucidated. Interestingly, no significant differences in abundancy and distribution of smooth muscle cells have been found in antibiotic-treated mice with respect to wild type. On the contrary a marked reduction in macrophage-expressed VEGFC was found in antibiotic-treated mice, together with their abundance. Moreover, this reduction in macrophage content was inhomogeneous along the small intestine but followed the normal density distribution of microbiota, being more affected in jejunum and ileum, and marginally present in the duodenum. Knockout mice carrying a depletion in CX3CR1^+^ macrophages displayed a marked reduction in VEGFC mRNA in both jejunum and ileum, confirming this mechanism [80]. Overall, it seems very likely that gut microbiota can control the number of macrophages in the villus, and in turn, this causes a different level of VEGFC that controls lacteals sprout and stabilisation in adult mice (Figure 2). At the macrophage level, the receptor family involved in microbiota sensing appears to be TLR (Toll-Like Receptor), and, by means of MyD88 signalling [98], this activation stimulates the production of VEGFC, since MyD88-depleted mice closely resemble GF or antibiotic-treated mice.

## 7. Closing Remarks

Lymph formation and propulsion are crucial to attain the correct fluid homeostasis of interstitial tissue and serosal cavities. In the peculiar gut microenvironment, this primary requirement is intertwined with the need of lipid transport associated with the absorption of dietary lipids, and the compartmentalised immunosurveillance exerted by DCs recirculating between the villi interstitial space and mesenteric lymph nodes. All these factors are mutually coordinated and any small imbalance, in the short or medium time frame, can cause severe illness due to oedema, reduced dietary lipids transport to the blood or even lack of immune surveillance. Since the late 1980s and 1990s, it has been clear that gut-derived lymph was accountable for lung and disseminated organ failure, leading to critical illness and death. However, limiting or abolishing the gut-derived lymph flow could not be a solution, since fluid homeostasis and lipid transport would be affected as well, with added morbidities. Most of the research in recent years has been focused on the primary site of potential translocation of bacteria, bacterial-derived or even tissue-derived toxins to the lymph, trying to unveil possible sites of intervention at the first step of this potentially life-threatening process. Nowadays, as witnessed by the findings summarised in this review, we have gained a very detailed (but still incomplete) view on the effect of gut microbiota on the development and stability of lacteals, whereas the knowledge on the reverse relationship between lymphatic function and gut microbiota is still to be acquired. Only recently scant data of a possible modulation of gut microbiota by the extent of lymph drainage have been observed. However, the possibility that an artificially imposed lymph flow could improve the composition of microbiota and, in doing so, prevent the severe consequences of gut-derived critical illness might foster a new wave of research in this direction.

## Data Availability

Not applicable.
